# Participation of children and young people with cerebral palsy in activities of daily living in rural Uganda

**DOI:** 10.1111/dmcn.15323

**Published:** 2022-06-26

**Authors:** Carin Andrews, Lukia Hamid Namaganda, Christine Imms, Ann‐Christin Eliasson, Elizabeth Asige, Godfrey Wanjala, Angelina Kakooza‐Mwesige, Hans Forssberg

**Affiliations:** ^1^ Department of Women's and Children's Health Karolinska Institutet Stockholm Sweden; ^2^ CURIE Study Consortium Iganga‐Mayuge Health and Demographic Surveillance System Iganga Uganda; ^3^ Department of Epidemiology and Biostatistics Makerere University Kampala Uganda; ^4^ Department of Paediatrics The University of Melbourne Melbourne VIC Australia; ^5^ Neuropaediatric Unit Astrid Lindgren Children's Hospital Stockholm Sweden; ^6^ Department of Pediatrics and Child Health Makerere University Kampala Uganda

## Abstract

**Aim:**

To compare the participation attendance and involvement of children and young people with and without cerebral palsy (CP) in a low‐resource area of Uganda.

**Method:**

Eighty‐two children and young people with CP aged 6 to 22 years (49 males, 33 females) and 81 age‐ and sex‐matched peers without CP (6 to 22 years; 48 males, 33 females) participated in this population‐based, cross‐sectional study. Data on attendance and involvement in 20 home and community activities were obtained using Picture My Participation, an instrument intended to measure participation in children with disabilities, particularly in low‐ and middle‐income countries. Non‐parametric statistical methods were used to assess between‐group differences. Effect size estimates were calculated.

**Results:**

Pooled attendance across all activities was lower in children and young people with CP than in children and young people without CP (*p* < 0.001) and for each activity item (*p* = 0.004 to *p* < 0.001). The effect sizes for each activity were 0.2 to 0.7. Between‐group differences were larger for community activities than for home activities. Pooled involvement across all activities was less in the group with CP (*p* < 0.001) and for each activity (*p* = 0.014 to *p* < 0.001). The effect sizes for each activity were 0.2 to 0.5. Children and young people in Gross Motor Function Classification System (GMFCS) levels I and II had higher attendance (*p* < 0.001) and involvement (*p* = 0.023) than those in GMFCS levels III to V.

**Interpretation:**

Participation of young people living with CP in Uganda was restricted, especially for community activities. There is a need to identify context‐specific participation barriers and develop strategies to overcome them.

**What this paper adds:**

- Children and young people with cerebral palsy (CP) attended all activities less than their peers without CP.
- Differences in attendance were larger for community‐based activities than home activities.
- When attending activities, children and young people with CP were less involved than their peers.
- Children and young people with milder impairments attended less frequently than their peers without CP.
- Children and young people with milder impairments attended more frequently than their peers with severe impairments.

## What this paper adds

Participation of children and young people with cerebral palsy in Uganda is immensely restricted compared to their peers, in particularly in community activities and especially for those with severe motor impairments.

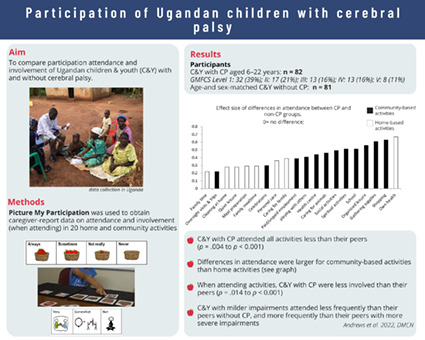

Video Podcast: https://youtu.be/cTOKIrKvvKQ

This original article is commented on by Kija on pages 155–156 of this issue.

**Abbreviation**
PMP: Picture My Participation.

Participation is a human right endorsed by the United Nations for all children, including those with disability.^1^, ^2^ Increased participation can both protect and promote health and well‐being, and it is often the ultimate goal of health interventions.^3^ Through participation, children engage in their communities, learn the skills and abilities required to navigate the contexts and situations in which they live, build a sense of their own capacities and preferences, and develop relationships.^4^ The International Classification of Functioning, Disability and Health framework has influenced the perspective on disability over the past decades to shift the focus from impairments to participation and emphasizing the need to understand and address environmental factors.^3^

Few studies have been published examining the participation of African children with cerebral palsy (CP); most studies have focused on ‘body organ and function’.^5^ A recent review on the participation of young people with disability identified only three studies from low‐income countries and 18 studies from low‐ and middle‐income countries in Africa.^6^ In contrast, substantial literature exists on the participation of children with CP in high‐income countries, showing that children with CP and other disabilities participate less often than their peers and are more likely to participate with family members in settings close to home.^7^ Because socioeconomic and cultural contexts influence participation in everyday activities,^8^ participation patterns may differ across sub‐Saharan Africa.^9^ In this region, children with disability often live in poor conditions with scarce access to health, education, and social services.^10^, ^11^ In addition, lack of knowledge, prejudice, and cultural norms can lead to stigmatization, discrimination, and profound barriers to participation in social activities.^9^, ^12^

To identify the need and targets for participation‐focused interventions for children and young people with disabilities, a detailed understanding of their patterns of participation is required, along with a knowledge of local contexts. We have previously studied a population‐based cohort of children and young people with CP in a rural region of Uganda, reporting on epidemiology, mortality, functional limitations, and access to services and education.^11^, ^13^, ^14^, ^15^ We found a 1.5 to 2.0 times higher prevalence of CP; these children had extremely low access to habilitation services and assistive devices and had a low participation rate in education. In addition, the children with CP in this cohort had excessive premature mortality, 25 times higher risk than the general population, due to malnutrition and infectious diseases. In this study, we aimed to describe their participation. We defined participation as attendance and involvement in activities of daily living.^8^ Attendance is an objective measure of being present in a situation. It is commonly considered from the perspective of how frequently individuals attend specific activities or situations or their patterns of attendance across a range of activities. Involvement is the subjective experience of participation while attending. It is a more complex construct to measure; in this study, it is considered to encompass aspects of persistence, motivation, social connection, and affect while attending activities.^8^ We explored three sets of questions: (1) How frequently do children and young people with and without CP attend activities of daily living? Is there a difference between groups and is this difference greater for certain activities? (2) Does sex, age, or impairment severity influence the frequency of attendance? (3) What is the level of involvement of children and young people with and without CP in the activities they attend? Is there a difference between groups?

## METHOD

### Study design and setting

This was a population‐based, cross‐sectional study of children and young people with and without CP living in the Iganga‐Mayuge Health and Demographic Surveillance System, which includes more than 80 000 inhabitants in 65 villages in rural eastern Uganda. This community consists of predominantly rural subsistence farmers, with more than 60% living below the poverty level.^15^ Having a child with a disability in this region is traditionally believed to be a punishment for wrongdoing, such as involvement in an extramarital affair, or caused by witchcraft, demons, or evil spirits.^9^ These beliefs instigate shame and stigma and lead to children being hidden in houses (Kakooza et al., unpublished material). Many families seek help from traditional healers; when children are not cured, they turn to health facilities which have limited knowledge of how to care for children with disabilities and health workers often show discriminatory attitudes.^11^ A typical home environment consists of a cluster of houses with poor entry access. The main entry door is often raised and the courtyard terrain is a mixture of loam soil and rough stones. Many of the caregivers spend a lot of their time each day attending to their crops, while their child either stays alone in a room or is placed in a secluded hut.

This study was approved by the Higher Degrees Research and Ethics Committee of the School of Public Health, College of Health Sciences, Makerere University, and the Uganda National Council for Science and Technology (reference no. HS 1734). All caregivers gave written informed consent to participate.

### Participants

Eighty‐two children and young people with CP aged 6 to 22 years and 81 age‐ and sex‐matched children and young people without CP participated. Participants were identified in a three‐stage screening in 2015, in which 31 756 children in the Iganga‐Mayuge Health and Demographic Surveillance System between 2 and 18 years took part in (1) door‐to‐door screening, (2) follow‐up by a trained CP team, and (3) clinical neurological assessment using the definition of CP by the Surveillance of CP in Europe.^11^, ^13^ At the 4‐year follow‐up in 2019, 15 of the 97 original children and young people with CP were deceased.^14^ The motor function, self‐care skills, and social functions of the surviving children and young people were assessed in 2019,^16^ along with the data collected for the current study. The characteristics of the children and young people and their relationship with their caregivers are presented in Table S1. There were no differences in the socioeconomic characteristics between households with children and young people with CP and those without CP except for marital status, with more caregivers being married in the group without CP. In both groups, more than two‐thirds of households were living below the Ugandan national poverty level (US$0.88–US$1.04 per day). The detailed functional and clinical information of the group with CP has been reported previously.^11^, ^13^, ^16^

### Assessments

Picture My Participation (PMP), an instrument intended to measure participation in children with disabilities, measures participation in 20 home and community activities in children and young people aged 5 to 21 years.^17^ Developed for use in low‐ and middle‐income countries, PMP is administered as a picture‐supported interview; each item and response category is illustrated by pictures. PMP can be administered either as a child or a parent/caregiver interview.^18^ It consists of four sections that measure attendance, involvement, importance, and facilitators/barriers. Results from the first two sections are reported in this study, while the results of the other two sections will be reported in a subsequent article.

Attendance frequency was recorded using a 4‐point Likert scale for each of the 20 activities: 1 = always, 2 = sometimes, 3 = seldom, or 4 = never. The different responses were illustrated with baskets containing different amounts of fruit (from a full to an empty basket) and respondents were instructed to point to the appropriate basket. Respondents were subsequently asked about the child's involvement in those activities, scored as ‘always’ or ‘sometimes’ for attendance. Involvement was rated on a 3‐point Likert scale: 1 = very, 2 = somewhat, or 3 = not. These alternatives were also illustrated with pictures: a child playing with other children, a child looking at other children when playing, and a child remaining by themself respectively.

Caregivers were interviewed using the PMP on behalf of their children and young people. Before using the PMP in this study, it was pretested on five caregivers of children and young people living in an area close by the study area to determine whether the procedure was acceptable and if the images were suitable for the community. PMP was designed to be a picture‐supported interview about participation in daily life, with the interview conducted as a conversation to explore participation in daily life and understand the child's experience. PMP is introduced to caregivers as ‘we want to understand your and your child's story…’. The pretest demonstrated that the response options were easily understood and led to some minor adjustments of the wording to better suit the Ugandan context.

**Figure 1:**
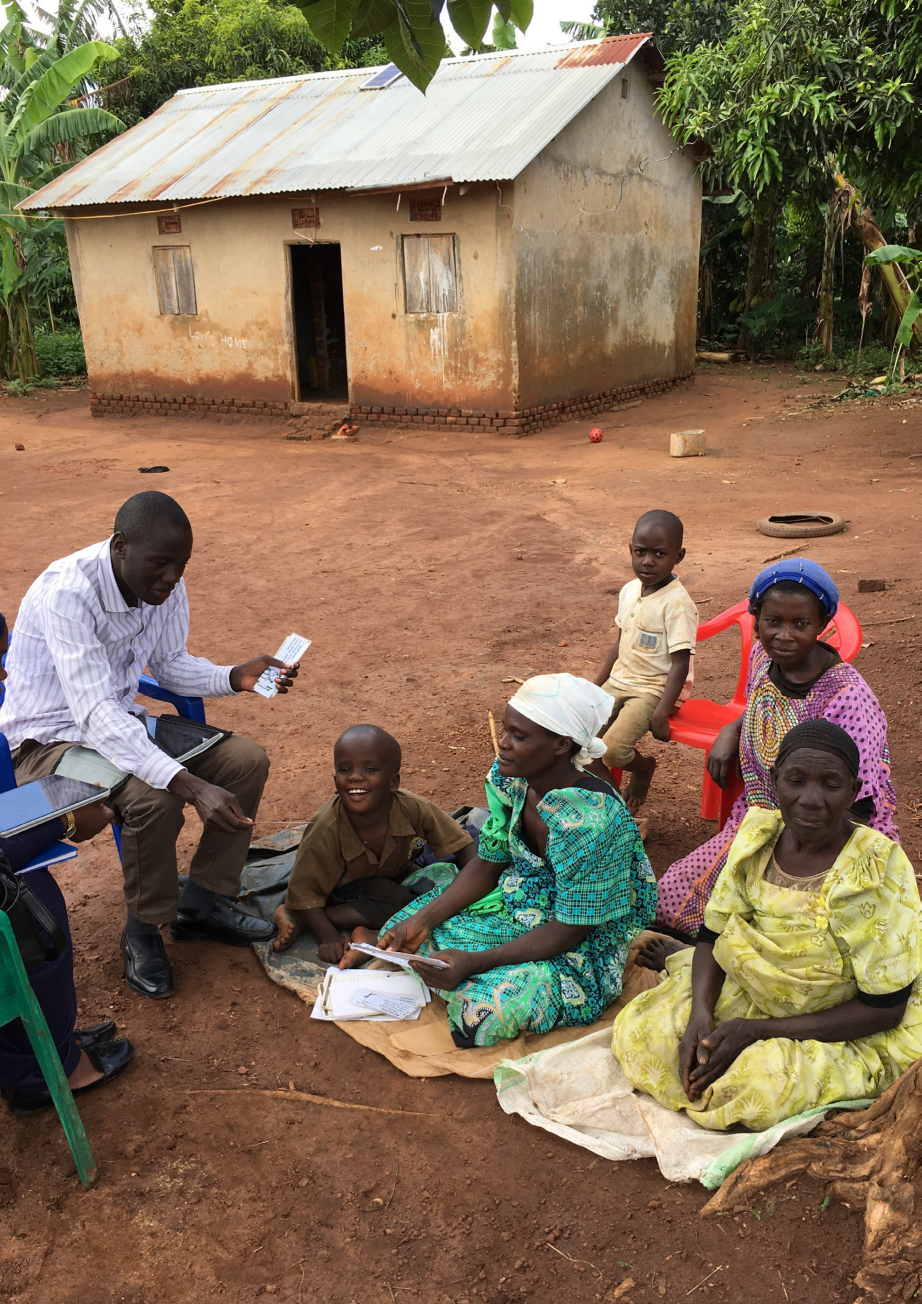
Picture My Participation interview with mother, child, and family outside the family home.

Caregiver perspectives were sought for consistency of reporting across all participants since approximately one‐half of children and young people with CP were unable to comprehend and respond accurately because of severe cognitive or communication impairments.^11^ The interviews were conducted by a social worker living in the area and familiar with the culture and language. The social worker received study‐specific training to ensure consistency and quality of data. The interview was performed in the home environment, typically under the shade of a tree, with the child surrounded by the caregiver and other household members (Figure 1).

### Procedure for data collection

PMP was delivered in the local language (Lusoga) to a caregiver who knew the child very well. The purpose of the test was introduced and the pictures for responding to attendance and involvement were explained. Practice items were used to teach the Talking Mats procedure that was used.^19^ Talking Mats is a visual communication method designed to support people to talk about what they know and think. Practice items were followed by an explanation of the attendance and involvement images that are used to indicate levels of participation. Because PMP is a process of talking about their usual daily lives and sharing ideas in a conversation, caregivers could draw on any information they had about their child's attendance and involvement to inform their response choices.

The interview, including all four sections, lasted 30 to 40 minutes. All responses were recorded electronically with tablets using the Open Data Tool Kit and data entry was reviewed for completeness and quality while still in the field.

The functional limitations of children and young people with CP were described using the Gross Motor Function Classification System (GMFCS).^16^ The GMFCS classifies mobility on a 5‐point ordinal scale, from level I (mild/independent) to level V (severe/dependent on assistance for all mobility functions). Children in GMFCS levels I and II were independent walkers, while children in levels III to V could not walk independently. GMFCS was assessed by therapists at a health centre close to the person's home within 1 to 2 weeks of the interview.

### Statistical analysis

We report our findings according to activity item and pooled proportions across all activity items. Activities were classified as predominantly home‐based (‘home’) or predominantly community‐based (‘community’) by the authors with experience of the local context (Appendix S1). For attendance and involvement, pooled proportions (combined data for all 20 activities) were calculated for each response category and presented as a percentage for each group as follows. For attendance, the total number of responses was 20 activities multiplied by the number of participants in the group. To calculate the percentages, the sum of responses in each category was divided by the total number of responses of the group and multiplied by 100. For attendance, the pooled proportions of involvement were calculated only for activities scored as ‘always’ or ‘sometimes’.

Spearman's rank correlation and *χ* ^2^ tests were used to evaluate differences in pooled proportions of attendance and involvement between groups with and without CP, as well as within‐group differences according to age (6–9 years, 10–14 years, 15–22 years), sex, and GMFCS level (I and II vs III–V). A Mann–Whitney *U* test was used to investigate differences between groups for attendance and involvement in each activity, with results presented as z‐scores and probability scores. Effect size (*r*) was calculated according to the formula $r=\frac{z}{\sqrt{N}}$ with *N* representing the total number of participants in both groups. Effect sizes were considered small when *r* was lower than 0.3 and large when *r* was greater than 0.5. Stata version 14.2 (StatCorp, College Station, TX, USA) was used for the statistical analyses. Because we undertook a range of analyses on this data set, we interpreted the strength of the evidence as being significant if *p* <0.01.

## RESULTS

### Attendance

#### Between‐group comparisons of pooled proportions

**Figure 2:**
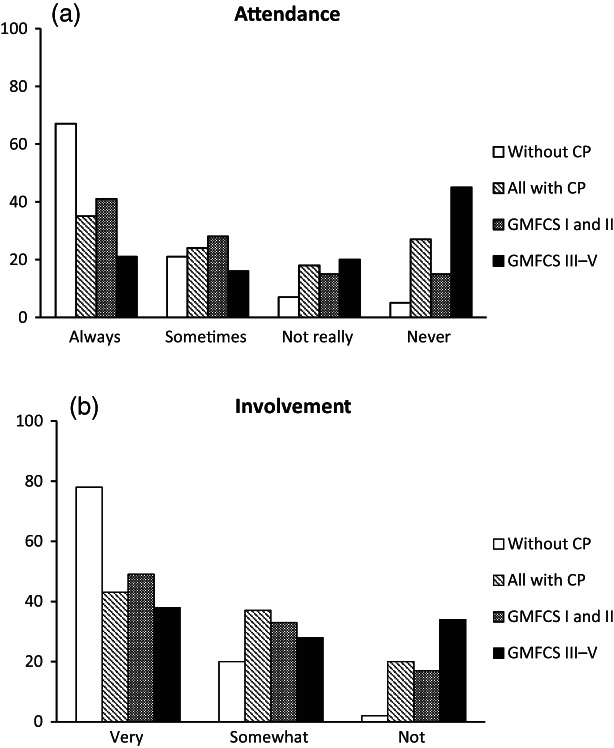
(a) The pooled proportions of attendance ratings for all 20 activities are presented for each response category (always, sometimes, not really, never). (b) The pooled proportions of involvement in the activities that participants attended always or sometimes are presented for each response category (very, somewhat, not). Data are presented as the percentage for all children and young people without cerebral palsy (CP), all children and young people with CP, and the group of children and young people with CP divided into two groups: those in Gross Motor Function Classification System (GMFCS) levels I and II and those in GMFCS levels III to V (for details, see ‘Method’).

Pooled proportions of attendance ratings for each response category (always, sometimes, not really, never) are shown in Figure 2a. Pooled attendance ratings of always or sometimes were observed in 57% of children and young people with CP and 87% of those without CP (df = 3, *χ* ^2^ = 28.71, *p* < 0.001). Pooled attendance ratings of always or sometimes were 70% for children and young people in GMFCS levels I and II and 35% for those in GMFCS levels III to V (df = 3, *χ* ^2^ = 27.91, *p* < 0.001). Children and young people in GMFCS levels I and II had significantly lower attendance than children and young people without CP (df = 3, *χ* ^2^ = 13.50, *p* = 0.004).

#### Between‐group comparisons for each activity

**Figure 3:**
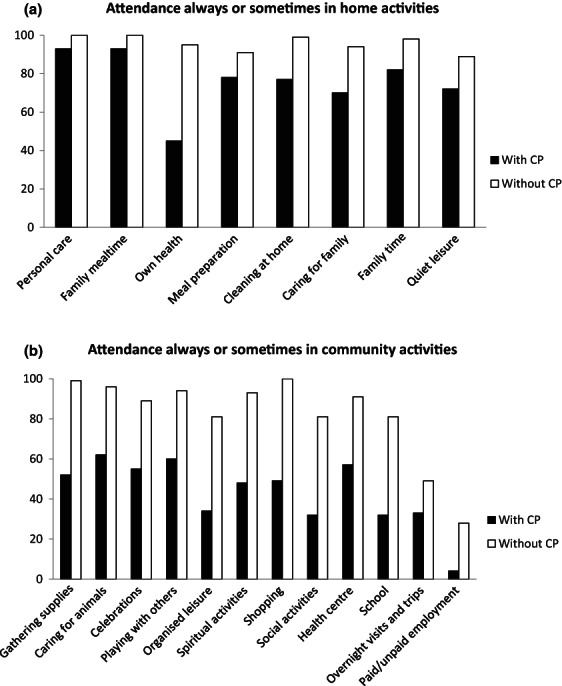
Attendance of the group with (black) and the group without cerebral palsy (CP) (white) in home (a) and community activities (b). The percentage of children and young people attending always or sometimes in each of the 20 activities is shown.

Almost all children and young people without CP attended most activities always or sometimes, except overnight visits and trips and paid/unpaid employment (Table 1 and Figure 3). Children and young people with CP attended fewer activities always or sometimes and had lower attendance for all activities (*p* ≤ 0.004). When activity items were categorized as home or community, children and young people with CP had visibly lower attendance for community activities (Figure 3b).

**Table 1:** Attendance in each of the 20 activities categorized as always, sometimes, not really, and never in the groups with (*n* = 82) and without cerebral palsy (CP) (*n* = 81)

Activity item in the PMP	Always		Sometimes		Not really		Never		z‐score	*p*	Probability score	*r*
	Without CP, *n*	With CP, *n*	Without CP, *n*	With CP, *n*	Without CP, *n*	With CP, *n*	Without CP, *n*	With CP, *n*				
Personal care	80	61	1	15	0	3	0	3	4.55	0.000	0.62	0.36
Family mealtime	76	59	5	17	0	5	0	1	3.76	0.000	0.61	0.29
Own health	67	14	10	23	4	29	0	16	8.56	0.000	0.86	0.67
Gathering supplies	64	19	16	24	1	9	0	30	7.83	0.000	0.83	0.61
Meal preparation	53	31	21	33	7	10	0	8	3.72	0.000	0.65	0.29
Cleaning at home	57	40	23	23	1	9	0	10	3.52	0.000	0.64	0.28
Caring for family	63	35	13	22	5	15	0	10	4.91	0.000	0.70	0.39
Caring for animals	68	35	10	16	3	12	0	19	5.90	0.000	0.73	0.46
Family time	63	49	16	18	2	14	0	1	2.85	0.004	0.61	0.22
Celebrations	35	25	37	20	8	18	1	19	3.79	0.000	0.66	0.30
Playing with others	55	28	21	21	5	14	0	19	5.27	0.000	0.72	0.41
Organized leisure	42	9	24	19	12	17	3	37	7.09	0.000	0.81	0.56
Quiet leisure	60	39	12	20	8	15	1	8	3.62	0.000	0.64	0.28
Spiritual activities	53	20	22	19	5	15	1	28	6.49	0.000	0.78	0.51
Shopping	70	23	11	17	0	9	0	33	8.05	0.000	0.83	0.63
Social activities	24	9	42	17	12	24	3	32	6.30	0.000	0.78	0.49
Health centre	49	19	25	28	6	29	1	6	5.59	0.000	0.74	0.44
School	64	23	2	3	0	1	15	55	6.51	0.000	0.76	0.51
Overnight visits and trips	21	10	19	17	26	25	15	30	2.86	0.004	0.63	0.22
Paid/unpaid employment	13	1	10	2	12	5	46	74	4.97	0.000	0.67	0.39

Abbreviation: PMP, Picture My Participation.

A Mann–Whitney *U* test was used to investigate the difference between groups for each activity.

**Figure 4:**
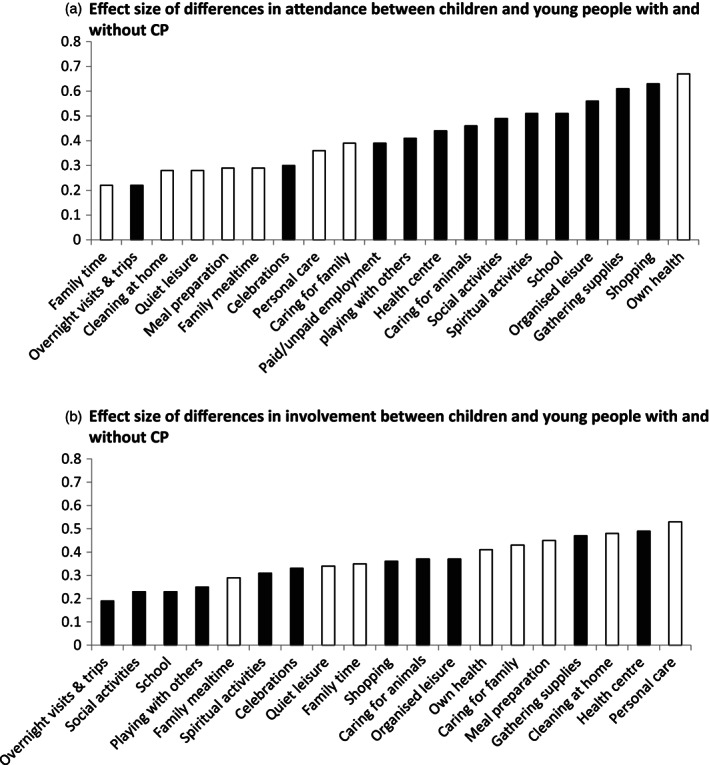
The effect size of the difference in attendance (a) and involvement (b) between children and young people with cerebral palsy (CP) and children and young people without CP in activities is presented in increasing order. Home activities are coloured white and community activities are coloured black. For involvement, only 19 activities were analysed.

Effect sizes for between‐group differences in attendance ranged from 0.22 to 0.67 for each activity, with large effects for six activities (Table 1 and Figure 4a).

#### Sex and age differences

There were no differences in attendance according to sex or age in any group (*p* > 0.05). Correlation between age and the number of activities attended was low in both groups (*r* = 0.13 for children and young people with CP, −0.10 for their peers without CP).

### Involvement

#### Between‐group comparisons of pooled proportions

Pooled proportions of involvement ratings for the activities were only calculated for activities in which the respondents attended always or sometimes: these are presented for both groups in Figure 2b. Most children and young people without CP (78%) were very involved when attending activities; only a few were not involved. For children and young people with CP, fewer than 50% were very involved and 20% were not involved in the activities they attended, providing strong evidence of between‐group differences (df = 2, *χ* ^2^ = 29.92, *p* < 0.001). Children in GMFCS levels I and II were more involved than children in GMFCS levels III to V (df = 2, *χ* ^2^ = 7.46, *p* = 0.023; Figure 2b).

#### Between‐group comparisons for each activity

The number of participants attending each activity varied from 23 to 81 in the group without CP and from 3 to 76 in the group with CP (Table 2). The number of activities rated as very involved for more than 50% of participants was two in the group with CP and 15 in the group without CP. There was strong evidence of differences between groups for 19 activities, with higher proportions of the group without CP being involved than the group with CP (*p* ≤ 0.014).

**Table 2:** Involvement in each of the 20 activities categorized as very, somewhat, and not in the groups with and without cerebral palsy (CP)

Activity item in the PMP	Very		Somewhat		Not		Total		z‐score	*p*	Probability score	*r*
	Without CP, *n*	With CP, *n*	Without CP, *n*	With CP, *n*	Without CP, *n*	With CP, *n*	Without CP, *n* ^a^	With CP, *n*				
Personal care	81	42	0	16	0	18	81	76	6.73	0.000	0.72	0.53
Family mealtime	80	62	1	4	0	10	81	76	3.68	0.000	0.59	0.29
Own health	57	9	17	19	3	9	77	37	5.20	0.000	0.77	0.41
Gathering supplies	76	21	4	20	0	2	80	43	5.97	0.000	0.73	0.47
Meal preparation	56	19	18	32	0	13	74	64	5.79	0.000	0.76	0.45
Cleaning at home	65	21	15	30	0	12	80	63	6.09	0.000	0.76	0.48
Caring for family	54	17	18	15	4	25	76	57	5.46	0.000	0.75	0.43
Caring for animals	63	22	14	18	1	11	78	51	4.71	0.000	0.71	0.37
Family time	70	38	9	22	0	7	79	67	4.49	0.000	0.67	0.35
Celebrations	37	9	31	21	4	15	72	45	4.26	0.000	0.72	0.33
Playing with others	62	27	13	19	1	3	76	49	3.23	0.001	0.64	0.25
Organized leisure	35	3	27	13	4	12	66	28	4.72	0.000	0.79	0.37
Quiet leisure	59	28	13	26	0	5	72	59	4.29	0.000	0.68	0.34
Spiritual activities	59	17	16	19	0	3	75	39	3.91	0.000	0.68	0.31
Shopping	76	24	5	14	0	2	81	40	4.64	0.000	0.67	0.36
Social activities	24	4	39	15	3	7	66	26	2.91	0.004	0.67	0.23
Health centre	60	13	14	19	0	15	74	47	6.32	0.000	0.80	0.49
School	55	14	11	12	0	0	66	26	2.93	0.003	0.65	0.23
Overnight visits and trips	30	13	9	9	1	5	40	27	2.46	0.014	0.65	0.19
Paid/unpaid employment	7	1	6	2	10	0	23	3	–	–	–	–

Abbreviation: PMP, Picture My Participation.

^a^ The number of responders for each activity item is shown for each group. Involvement was only measured in activities that the participant attended always or sometimes. A Mann–Whitney *U* test was used to investigate difference between groups for each activity. −, no analysis was performed for the paid/unpaid employment activity due to low frequency of attendance in both groups.

Effect sizes for between‐group differences in involvement ranged from 0.19 to 0.53 for each activity, with large size effects for two activities (Table 2 and Figure 4b).

## DISCUSSION

Our main finding is that children and young people with CP in a resource‐limited region of Uganda participated less than children and young people without CP across 20 activities of daily living. Our analyses revealed significant differences between groups in both attendance and involvement for all activities. The largest differences in attendance occurred in community activities, while differences were smaller for home‐based activities. In addition, children and young people with more severe limitations in gross motor function had lower levels of both attendance and involvement, while attendance and involvement were not associated with age or sex.

The restricted attendance of Ugandan children and young people with CP, compared with their peers without CP, was expected and consistent with the results of studies from high‐income countries.^4^, ^7^, ^20^, ^21^ Nevertheless, the range and magnitude of this restriction, with highly significant differences for all 20 activities, was dramatic. The few studies about participation of children with disability from low‐ and middle‐income countries in Africa mostly involved children with intellectual disability.^6^ A recent study from South Africa showed less frequent attendance for children with intellectual disability than for those without in only 9 of 20 PMP activities. The authors concluded that there were more similarities than differences between groups regarding attending everyday activities, in line with the results of studies in children with intellectual disability across low‐ and high‐income contexts.^22^ This contrasts with our findings that the entire spectrum of activities is affected. The discrepancy may be attributed to different cultural and economic settings or the possibility that children with CP are more restricted than children with intellectual disability. The study from South Africa was performed in an urban area in Pretoria and involved children who attended school in English and who had mild‐to‐moderate intellectual disability but no hearing, visual, or motor impairments. In contrast, our population‐based cohort of children and young people with CP was located in a rural area and most participants did not attend school. A large proportion were non‐verbal and non‐ambulatory and had severe concurrent disorders, including epilepsy.^11^, ^16^ Thus, the different study results could be explained by differences in socioeconomic demographics, impairment type and severity, and participant selection. Recruiting participants from schools, as in the study from South Africa, may not provide a representative sample of children with intellectual disability. Our results support the effects of mobility limitations on participation. Studies from high‐income countries suggested that diagnosis itself is not as important to participation as level of functioning and that factors other than disability, such as age, sex, socioeconomic opportunities, and geographical region, may play a role.^22^, ^23^, ^24^, ^25^ In this study, we found no differences according to age or sex.

The 20 PMP activities were selected to cover the range of activities in which children typically participate selected by reviewing existing measures and the activities and participation chapters of the International Classification of Functioning, Disability and Health.^17^ Our finding that most children and young people without CP participated in these activities supports their validity. The medium‐to‐large differences between groups in frequency of attendance in every activity highlights the impact of CP. The largest differences between groups were in community‐based activities, such as shopping, gathering supplies, organized leisure, school, and spiritual activities, with smaller differences in home‐based activities, including family time, cleaning at home, quiet leisure, meal preparation, family mealtime, and celebrations. These results suggest that factors outside the family play an important role in participation. A similar pattern between community and family activities was described for children with intellectual disability in South Africa^22^ and in some studies from high‐income countries.^26^, ^27^, ^28^ Being able to attend the full spectrum of activities deemed important to the individual, family, and sociocultural group is crucial because these activity settings provide opportunities for learning and development across all aspects of life.^29^

Another important finding of this study is that children and young people with CP were less involved in the activities they attended, further highlighting their restricted participation. Involvement is a complex concept, which can be understood from different perspectives.^8^ One reason for lower involvement might be less engagement from, and lower expectations of, the people around these children. Another reason could be the interaction between child‐related factors, making it more difficult and time‐consuming to involve the child, and the presence (or absence) of environmental supports, including people available to provide assistance. Our cohort of children and young people with CP included many children with severe motor and associated impairments,^11^ which restricted their ability to be involved. Unlike with attendance, we observed no clear pattern of reduced involvement related to whether activities were performed at home or in the community. The most severely impaired children, with sensory, cognitive, and communication impairments, require considerable assistance from caregivers, family members, and others to participate in essentially all activities of daily living. Reduced involvement in family activities may reflect the many competing tasks of Ugandan caregivers, such as caring for large extended families or spending long hours in the fields to ensure an adequate food supply. These limit the time available to spend with the child with impairments.

In this resource‐limited region, caring for a child with a disability is a financial burden, with substantial costs related to transportation, therapy, and assistive devices. Low participation in community activities could reflect a lack of appropriate assistive devices necessary to overcome long distances and other physical barriers in children with mobility restrictions. Even more crucial in this part of the world might be the stigma and negative attitudes towards children with CP stemming from beliefs and perceptions about children with disabilities.^9^, ^30^ It is sometimes believed that these children should be hidden from other community members.^31^ This stigmatization may lead not only to explicit exclusion from community activities but also to self‐stigmatization and family stigmatization, resulting in social withdrawal or hiding.^12^

### Study strengths and limitations

This is the first study investigating participation in activities of daily living of children and young people with CP living in a rural, low‐resource area of Africa. It involved a population‐based cohort of children and young people with CP and an age‐ and sex‐matched group of children without CP living in the same area, providing more representative samples than studies based on cohorts recruited from clinical or school settings. Using PMP improved our understanding of participation beyond simply attendance by also assessing involvement. Our study confirmed that PMP captures important differences in both attendance and involvement of children and young people with CP, compared with their peers without CP, in a low‐resource setting.^17^ A major strength of PMP in this setting is its pictorial grading system. This is important because cultural differences can make numerical scales difficult to use.

A limitation was that the PMP has not been validated in Uganda; however, we piloted the PMP and made some minor adjustments of wording before the study. After the study, we found that some PMP activity items were less relevant. Few children and young people had experienced paid or unpaid employment, most had not participated in overnight trips and visits, and the school item was only relevant for school‐age children. Another limitation of the study was that we could not obtain information directly from the children and young people but interviewed caregivers on their behalf instead. Previous studies have shown limited agreement between reports from children and those of their caregivers.^18^ In addition, the relatively small sample size constrained our analyses. Nevertheless, the study was quite large in relation to previous studies on activity participation and sufficiently large to show important differences between groups.

## CONCLUSIONS

Uganda ratified the United Nations Convention on the Rights of the Child in 1990 and the United Nations Convention on the Rights of Persons with Disabilities in 2008. In addition, several legislative bodies, policies, and socioeconomic programmers enable participation on an equal basis with others. However, the results of this article highlight the continuing gap between laws, policies, and practice. Our findings illustrate a disconnect between the implementation of rights‐based policies and meeting the needs of children and young people with CP in real life. Children and young people with CP attended all activities less frequently than their peers and were less involved in the activities they did attend. Evidence about the underlying facilitators and barriers behind these patterns has been gathered and will be reported in a subsequent article. This additional knowledge is crucial for identifying culturally and context‐specific targets for change and developing interventions to enhance participation in everyday activities of children and young people with disability.

## Supporting information


**Appendix S1:** Comments on authorship.

**Table S1:** Sex and age of the children and young people with and without CP, and relationship to the caregiver who performed the PMP interview.

## Data Availability

The data that underlie the results reported in this article are described at the Swedish National Data Service. Dataare made available upon request after ensuring compliance with relevant legislation. https://doi.org/10.5878/febd‐dx96
