# Supplementary material for: Participation of children and young people with cerebral palsy in activities of daily living in rural Uganda
Source: Dev Med Child Neurol. 2022 Jun 26;65(2):274–84. doi: 10.1111/dmcn.15323 (PMC10083931; doi:10.1111/dmcn.15323)
Supplement: Supplementary file 1 — Appendix S1: Comments on authorship. [file DMCN-65-274-s001.docx]

Supplement 1

Comment on the LMIC-HIC collaboration and authorship:

This paper is one in a series of 19 publications (16 published) originating from a long collaboration between academic teams at Makerere University College of Health Sciences led by Professor Angelina Kakooza Mwesige, and Karolinska Institutet (Professor Hans Forssberg) funded by the Swedish Research Council. The main focus of the collaboration has been to study children with Cerebral palsy (CP) in rural Uganda (CURIE-consortium; CP in Uganda: risk factors, intervention and epidemiology). A second goal has been to build local academic capacity in developmental disabilities in Uganda. Through the collaboration Dr Kakooza gained her PhD and later promotion to Associate Professor at Makerere University, Kampala. Two PhD students from Uganda have been appointed and are now working on different parts of the project; one is planning to complete her thesis next year. Two Masters students in the Department of Paediatrics and Child Health at Makerere University have published their theses on Cerebral palsy supervised by Prof. Kakooza. Several therapists and social workers have been employed and trained in evidence based, contemporary assessments and interventions. Professor Kakooza and her team now constitute one of the leading research centres in the field of childhood disability in Africa.

During the review process of this paper, concerns were raised regarding research parity between authors from high income countries (HICs) and low- and middle-income countries (LMICs), and it was noted that neither the first nor the last authors were from Uganda. This resonates with ongoing discussions on decolonisation on terms of parity and equity within Global Health Research and Global Health Partnerships. First and last authorship are often occupied by researchers from HICs since they get most visibility and credit. Recent systematic reviews on authorship in Global Health research show that African authors are underrepresented in these positions which perpetuate power differences between LMIC and HIC researchers, the latter often the source of funding and therefore in control of the research agenda.

In our collaboration we have been aware of the problem with researchers from HICs exploring researchers from LMICs and not contributing to building local capacity. We have seen several examples of this “neo-colonial abuse” in Uganda. Therefore, we have from the beginning of the collaboration considered the balance between the two partners reflected in the authorship of the series of 19 papers in which an author from a LMIC is either first or last author in 16 of the papers. At the same time, we have considered that the order of authorship reflects the role, engagement, responsibility and the work the author has carried out in that specific study. First or last authorship is important for the academic career and funding opportunities. It would not be right, for merely political reasons, to give these positions away. That would risk diluting these positions in studies originating from LMIC, which probably would give a backlash in a longer perspective.

Therefore, in order to build capacity, it has been important that researchers from Uganda or other LMICs have fulfilled the task to become first or last author (which has been the case in 84% of the studies). In this specific study the analysis was mainly done by the first and last authors, the last author drafted the manuscript while all authors were involved in discussing the results, outlining the introduction and discussion and reading and editing the manuscript.

The Makerere University- Karolinska Institutet research collaboration CURIE consortium began in 2014 and below are the publications from the team of Principal investigators Makerere University (A. Kakooza-Mwesige) – Karolinska Institutet (H. Forssberg) and co-investigators working on children and youth with developmental disabilities in rural Uganda. Authors from Uganda or other LMICs are in bold.

Papers published:

1. **Kakooza-Mwesige, A.**, H. Forssberg, A. C. Eliasson and **Tumwine J. K.** (2015a). "Cerebral palsy in children in Kampala, Uganda: clinical subtypes, motor function and co-morbidities." BMC Res Notes 8: 166.
2. **Kakooza-Mwesige, A., J. K. Tumwine,** A. C. Eliasson, **H. K. Namusoke** and H. Forssberg (2015b). "Malnutrition is common in Ugandan children with cerebral palsy, particularly those over the age of five and those who had neonatal complications." Acta Paediatr 104(12): 1259-1268.
3. **Kakooza-Mwesige, A., R. K. Byanyima, J. K. Tumwine**, A. C. Eliasson, H. Forssberg and O. Flodmark (2016). "Grey matter brain injuries are common in Ugandan children with cerebral palsy suggesting a perinatal aetiology in full-term infants." Acta Paediatr 105(6): 655-664.
4. **Kakooza-Mwesige A, Tumwine JK,** Forssberg H, Eliasson AC. The Uganda version of the Pediatric Evaluation of Disability Inventory (PEDI). Part I: Cross-cultural adaptation. Child Care Health Dev. 2018 Mar 12. doi: 10.1111/cch.12563. [Epub ahead of print] PMID: 29527735
5. **Amer A, Kakooza-Mwesige A,** Jarl G, **Tumwine JK**, Forssberg H, Eliasson AC, Hermansson L., The Ugandan version of the Pediatric Evaluation of Disability Inventory (PEDI-UG). Part II: Psychometric properties. Child Care Health Dev. 2018 Jul;44(4):562-571. doi: 10.1111/cch.12562. Epub 2018 Mar 13.
6. **Kakooza-Mwesige, A.**, (2016). Cerebral Palsy in Mulago Hospital, Uganda: co-morbidity, diagnosis and cultural adaptation of an assessment tool. Thesis for doctoral degree (PhD). Karolinska Institutet. ISBN 978-91-7676-208-0
7. **Kakooza-Mwesige A**, Andrews C, Peterson S, **Wabwire Mangen F**, Eliasson AC, Forssberg H. Prevalence of cerebral palsy in Uganda: a population-based study. Lancet Glob Health. 2017 Dec;5(12):e1275-e1282. doi: 10.1016/S2214-109X(17)30374-1. Epub 2017 Nov 5. PMID: 29102350
8. **Munyumu K, Idro R, Abbo C, Kaddumukasa M, Katabira E, Mupere E,** **Kakooza-Mwesige A.** Prevalence and factors associated with sleep disorders among children with cerebral palsy in Uganda; a cross-sectional study. *BMC Pediatr. 2018 Feb 5;18(1):26*
9. Andrews C, **Kakooza-Mwesige A**, Almeida R, Swartling Peterson S, **Wabwire-Mangen F**, Eliasson AC, Forssberg H., Impairments, functional limitations, and access to services and education for children with cerebral palsy in Uganda: a population-based study. Dev Med Child Neurol. 2019 Nov 25. doi: 10.1111/dmcn.14401. [Epub ahead of print] PMID: 31762018
10. **Namaganda LH**, Almeida R, **Kajungu D, Wabwire-Mangen F**, Peterson S, Andrews C, Eliasson AC, **Kakooza-Mwesige A**, Forssberg H., Excessive premature mortality among children with cerebral palsy in rural Uganda: A longitudinal, population-based study. PLoS One. 2020 Dec 29;15(12):e0243948. doi: 10.1371/journal.pone.0243948. eCollection 2020. PMID: 33373366
11. **Saloojee G, Ekwan F**, Andrews C, Damiano DL, **Kakooza-Mwesige A**, Forssberg H., Akwenda intervention programme for children and youth with cerebral palsy in a low-resource setting in sub-Saharan Africa: protocol for a quasi-randomised controlled study. BMJ Open. 2021 Mar 8;11(3):e047634. doi: 10.1136/bmjopen-2020-047634.PMID: 34006038
12. Andrews C, **Namaganda L**, Eliasson AC, **Kakooza-Mwesige A**, Forssberg H., Functional development in children with cerebral palsy in Uganda: population-based longitudinal cohort study. Dev Med Child Neurol. 2021 Aug 4. doi: 10.1111/dmcn.14996. Online ahead of print. PMID: 34346507
13. **Bambi EN,** **Kakooza-Mwesige A**, **Lekuya HM, Kasirye P, Idro R**. Chronic pain among children with cerebral palsy attending a Ugandan tertiary hospital: a cross-sectional study. BMC Pediatr. 2021 Oct 18;21(1):456.
14. **Amer A**, Hermansson L, Jarl G, **Kamusiime S**, Forssberg H, Andrews C, **Kakooza-Mwesige A**, Eliasson AC. Validity and test-retest reliability of the Ugandan version of the Pediatric Evaluation of Disability Inventory (PEDI-UG) in children and youth with cerebral palsy. Accepted for publication in Child: Care, Health & Development.
15. C Andrews, C Imms, AC Eliasson, **Wanjala G, Kansiime S, Opio E, Namaganda L, A Kakooza-Mwesige**, H Forssberg. Participation of children and youth with cerebral palsy in Uganda: Activity attendance and involvement compared with peers without cerebral palsy. Accepted for publication by DMCN.

Submitted manuscripts:

1. **Namaganda L**, Andrews C, **Wabwire-Mangen F**, Peterson S, Forssberg H, **Kakooza-Mwesige, A** Nutritional status and growth of children and youth with cerebral palsy in eastern Uganda: a longitudinal, comparative, population-based study. Final version Manuscript to be submitted

Manuscripts in preparation:

1. **Kakooza-Mwesige A, Atuyambe LM, Ssegujja E**, Andrews C, **Wabwire-Mangen F**, Eliasson AC, Forssberg H. Recognition of Cerebral Palsy and Understanding its Local Terminologies in rural Uganda: a community qualitative study. Manuscript
2. **Kakooza-Mwesige A, Atuyambe LM, Ssegujja E**, Andrews C, **Wabwire-Mangen F,** Eliasson AC, Forssberg H. Beliefs and practices regarding cerebral palsy and its management in children: perspectives from the community in rural Uganda. Manuscript.
3. **Kakooza-Mwesige A**, Kruer M, **Kansiime S, Opio E**, (XX collaborators TBD from Arizona University, USA), Andrews C, Forssberg H. Genetic and environmental risk factors for cerebral palsy in Uganda: a population-based study. Manuscript
