# Supplementary material for: Participation of children and young people with cerebral palsy in activities of daily living in rural Uganda
Source: Dev Med Child Neurol. 2022 Jun 26;65(2):274–84. doi: 10.1111/dmcn.15323 (PMC10083931; doi:10.1111/dmcn.15323)
Supplement: Supplementary file 2 — Table S1: Sex and age of the children and young people with and without CP, and relationship to the caregiver who performed the PMP interview. [file DMCN-65-274-s002.docx]

Supplemental Table 1

| **Characteristics of the CP- and non-CP groups** | | | |
| --- | --- | --- | --- |
|  | | CP (N=82)  N(%) | Non-CP (N=81)  N(%) |
| Sex | Male | 49 (60) | 48 (59) |
|  | Female | 33 (40) | 33 (41) |
| Age | 6-9 | 21 (26) | 30 (37) |
|  | 10-14 | 38 (46) | 25 (31) |
|  | 15-22 | 23 (28) | 26 (32) |
| Gross Motor Function Classification System (GMFCS) | I | 32 (39) |  |
|  | II | 17 (21) |  |
|  | III | 13 (16) |  |
|  | IV | 13 (16) |  |
|  | V | 8 (11) |  |
| Responding caregivers relationship to the child/youth | Mother | 43 (52) | 48 (59) |
|  | Father | 9 (11) | 13 (16) |
|  | Siblings | 12 (15) | 5 (6) |
|  | Other relative | 15 (18) | 10 (12) |
|  | Other | 3 (4) | 5 (6) |
| Head of household | Father | 48 (59) | 53 (67) |
|  | Step father | 5 (6) | 0 (0) |
|  | Mother | 10 (12) | 10 (13) |
|  | Grandmother | 9 (11) | 9 (11) |
|  | Other relative | 10 (12) | 7 (9) |
|  | Missing | 0 | 2 |
| Head of household highest education level | None | 10 (12) | 7 (10) |
|  | Primary | 50 (62) | 36 (51) |
|  | Senior | 16 (20) | 24 (34) |
|  | Post-secondary | 0 (0) | 3 (4) |
|  | Tertiary | 5 (6) | 1 (1) |
|  | Missing | 1 | 10 |
| Main source of Household income | Farming | 41 (50) | 40 (51) |
|  | Casual labour | 22 (27) | 21 (27) |
|  | Petty trade | 14 (17) | 13 (17) |
|  | Qualified labour | 5 (6) | 4 (5) |
|  | Missing | 0 | 3 |
| Estimated monthly household income in USD | <28 USD | 37 (47) | 32 (43) |
|  | 29-56 USD | 28 (36) | 27 (36) |
|  | 57-141 USD | 9 (12) | 13 (17) |
|  | >141 USD | 4 (5) | 3 (4) |
|  | Missing | 4 | 6 |

**Legend Supplemental Table 1:**

Sex, and age of the children and youth with and without CP, and the relation to the caregiver who performed the PMP interview and socio-economic data of the household. The GMFCS levels of children and youth with CP is presented. A detailed description of the functional and clinical data of the CP cohort is presented in Andrews et al 2020 and 2021 (see reference list).
